# A fungal spore calendar for England: Analysis of 13 years of daily concentrations

**DOI:** 10.1111/all.16356

**Published:** 2024-10-16

**Authors:** Fiona A. Symon, Samuel Anees‐Hill, Jack Satchwell, Abbie Fairs, Richard Edwards, Andrew J. Wardlaw, Leah Cuthbertson, Anna L. Hansell, Catherine H. Pashley

**Affiliations:** ^1^ Department of Respiratory Sciences University of Leicester Leicester UK; ^2^ Centre for Environmental Health and Sustainability University of Leicester Leicester UK; ^3^ Toxicology, UK Health Security Agency Harwell Campus Didcot UK; ^4^ NIHR Health Protection Research Unit in Environmental Exposures and Health at the University of Leicester Leicester UK; ^5^ NIHR Leicester Biomedical Research Centre Leicester General Hospital Leicester UK

To the Editor,

Fungal respiratory allergy affects up to 30% of hay fever sufferers and 70% of severe asthmatics in the UK.^1^ This study describes the seasonal patterns of airborne fungal spores found in central England.

Twenty‐three fungal spores were identified by microscopy in daily air samples collected in Leicester during the 13‐year period from 2007 to 2020 (excluding 2016) (Table S1). Details of methods are provided in Supporting Information. A spore calendar was constructed for the 9 most abundant spores (*Alternaria*, *Cladosporium*, *Didymella*, *Leptosphaeria*, *Sporobolomyces*, *Tilletiopsis* and *Ustilago*, *Aspergillus*/*Penicillium* type and coloured basidiospores) (Figure 1A). Of these, *Alternaria*, *Cladosporium* and *Aspergillus*/*Penicillium* are known allergens, while the remainder have been implicated in cases of allergy and thunderstorm asthma.^2^

**FIGURE 1:**
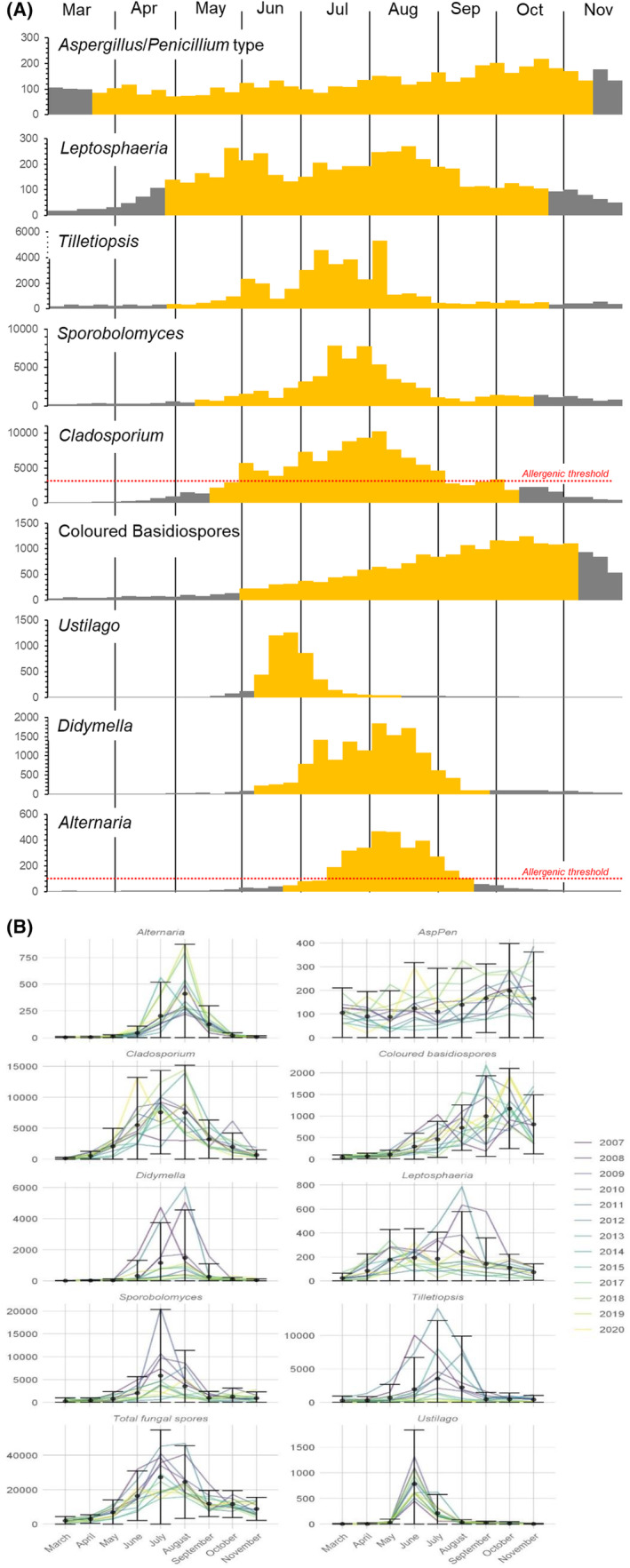
(A) Fungal spore calendar for the 9 main spores observed. Spores were ordered by the timing of their appearance. The main spore season (MSS) for each spore is shown in yellow (defined by the 90% method). Allergenic threshold concentrations for *Alternaria* and *Cladosporium* are indicated by the red dotted line. No allergenic threshold is available for any other spore type. Note the varying Y‐axis scales which represent spore concentration/m^3^/7‐days. (B) Annual distribution of spores over the 13 years sampled. Points display monthly means (spores/m^3^) with error bars displaying the standard deviation. Each year is represented by a single line.

All spore types showed a large degree of variability in total spore concentrations and daily maximal peak concentrations across the 13 years; however, temporal distributions were highly consistent over the study period (Figure 1B, Table S2). The spore season was bimodal, with the main peak period being in the summer months, coinciding with the greatest variety of species, followed by a smaller peak in autumn, mainly due to basidiospore release. Peak spore season showed a much slower decline than that of grass pollen, coinciding with the slow decline in antihistamine prescription (Figure 2A,B), suggesting that fungal spores may have a role in late summer hayfever as has been shown to be the case for *Cladosporium*.^3^

**FIGURE 2:**
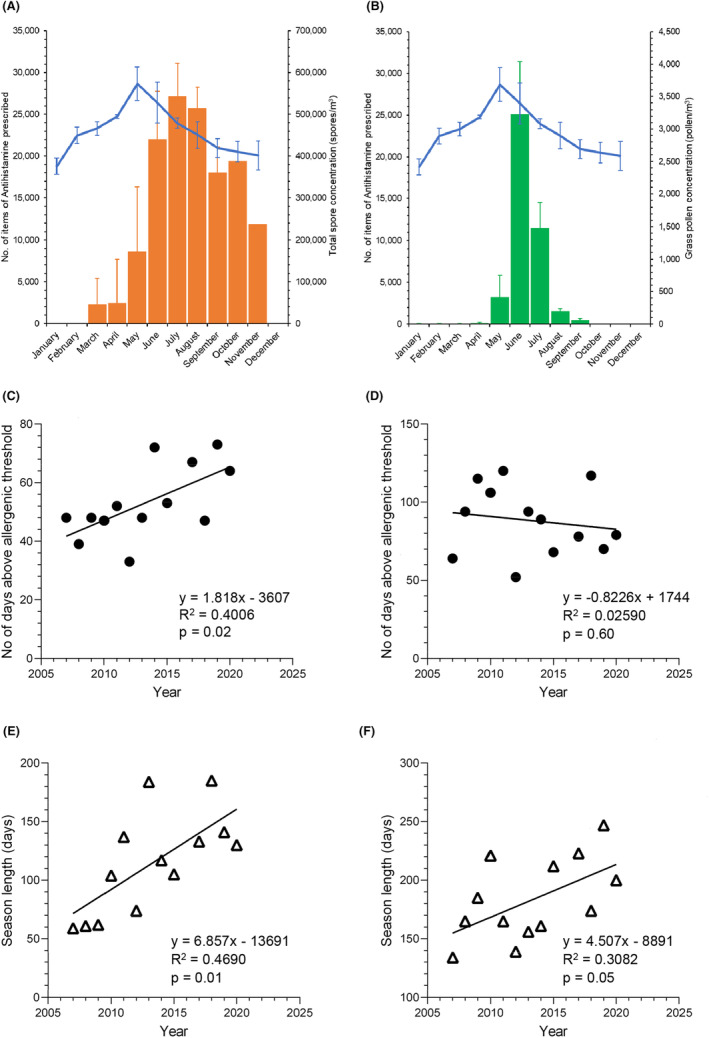
(A and B) Comparison of antihistamine prescribing (blue line) and (A) total fungal spore (orange bars) and (B) grass pollen (green bars) concentrations. Bars show the total fungal spore concentration or grass pollen concentration for each month, averaged for 2019–2023, the blue line shows the number of items of antihistamine prescribed by GPs in Leicestershire for the same time period.^7^ (C and D) Linear regression trend analysis for the number of days above allergenic threshold each year (closed circles) for (C) *Alternaria* and (D) *Cladosporium*. Allergenic threshold for *Alternaria* is >100 spores/m^3^/day, while that for *Cladosporium* is >3000spores/m^3^/day. (E and F) Linear regression trend analysis for the season length each year (open triangles) for (E) *Didymella* and (F) *Tilletiopsis*.

Considering individual fungal genera, *Ustilago*, a smut, was the first to reach its peak concentration in June, followed by *Cladosporium*, *Tilletiopsis and Sporobolomyces* which increased throughout June, peaking in July and August. *Alternaria* and *Didymella* levels were later to increase, rising in July and reaching their peak in August. Finally, coloured basidiospore concentrations increased slowly during the summer, not peaking until the autumn months. *Aspergillus*/*Penicillium* type and *Leptosphaeria* had both the earliest and longest spore seasons, but this combined with relatively low seasonal totals (Table S2) suggests a prolonged low‐level presence (Figure 1A).

Allergenic thresholds are only available for *Cladosporium* and *Alternaria* (>3000 and >100 spores/m^3^ per day, respectively).^4^ Over the study period *Cladosporium* spore levels exceeded allergenic threshold on 88 days per year on average (Table S2), while *Alternaria* exceeded published allergenic levels on 53 days. No correlation was observed between season length and the number of days above allergenic threshold for either spore.

The main meteorological factors influencing fungal spore concentration were temperature and precipitation.^5^ *Alternaria* and *Cladosporium* are “dry weather spores” and concentrations were significantly positively correlated to daily temperature (*p* < .01) (Table S3). The presence of *Didymella*, *Sporobolomyces*, *Tilletiopsis* and *Leptosphaeria* all correlated with precipitation (p < .01) consistent with their designation as “wet weather” spores.

The long‐term trend for total spore concentrations, over the 13‐year study, showed a statistically significant decrease (*p* = .03) due to significant decreases in *Sporobolomyces* and *Tilletiopsis* (*p* = .03 and .05, respectively), and decreases for *Didymella* and *Leptosphaeria*, which together make up four of the six most abundant spores considered in this study (Table S4). This decreasing trend for “wet weather spore” concentrations coincided with a significant decrease in daily precipitation (*R* ^2^ = 0.3234, *p* = .04). In contrast, *Alternaria* and *Cladosporium* totals showed an increasing trend, which reached statistical significance for *Alternaria* (*p* = .02) (Table S4). There were also significant increases in the number of days in which *Alternaria* exceeded allergenic threshold each year (*p* = .02) (Figure 2C–F) which may have clinical implications.

## CONCLUSIONS

This study provides a calendar for the seasonal distribution of the predominant airborne spores, in central England. There is a growing appreciation of the impact of fungal spores on health, with evidence to suggest that climate change will increase aeroallergen exposure.^6^ Long term aeroallergen monitoring sites such as at Leicester provide crucial information on trends in airborne fungal spore exposures, likely to have health implications.

## AUTHOR CONTRIBUTIONS

All authors reviewed and approved the final submitted version. F.A.S. acquired, analysed and interpreted the data, drafted the manuscript, revised the manuscript. S.H. analysed the data. J.S. acquired the data, revised the manuscript. A.F. acquired the data. R.E. acquired the data. A.J.W. revised the manuscript. L.C. revised the manuscript. A.H. revised the manuscript. C.H.P. Conceived and designed the study, revised the manuscript.

## FUNDING INFORMATION

This research was supported by National Institute for Health and Care Research (NIHR) Leicester Biomedical Research Centre (BRC), the Midlands Asthma and Allergy Research Association (MAARA) and the University of Leicester. AH acknowledges funding from the NIHR Health Protection Research Unit (HPRU) in Environmental Exposures and Health [grant number NIHR200901], a partnership between the UK Health Security Agency (UKHSA), the Health and Safety Executive (HSE) and the University of Leicester. CHP was also supported by the Academy of Medical Sciences. The views expressed are those of the author(s) and not necessarily those of the NIHR, UKHSA, Department of Health and Social Care, MAARA or the University of Leicester.

## CONFLICT OF INTEREST STATEMENT

The authors have no conflicts of interest to declare.

## Supporting information


Data S1.

## Data Availability

The data that support the findings of this study are available from the corresponding author upon reasonable request.
