# Supplementary material for: A fungal spore calendar for England: Analysis of 13 years of daily concentrations
Source: Allergy. 2024 Oct 16;80(2):617–20. doi: 10.1111/all.16356 (PMC11804307; doi:10.1111/all.16356)
Supplement: Supplementary file 1 — Data S1. [file ALL-80-617-s001.docx]

**A Fungal Spore Calendar for England: Analysis of 13 years of Daily Concentrations**

Fiona A. Symon, PhD^a^, Samuel Anees-Hill, PhD^b,c,d^, Jack Satchwell, BSc^a^, Abbie Fairs, PhD^a^, Richard Edwards, BSc^a^, Andrew J. Wardlaw MD^a^, Leah Cuthbertson, PhD^a^, Anna Hansell, MD, PhD^b,d,e^ and Catherine H. Pashley, PhD^a^.

**Affiliations:**

a Department of Respiratory Sciences, University of Leicester, University Road, Leicester, LE1 7RH ([fas4@leicester.ac.uk](mailto:fas4@leicester.ac.uk), [js660@leicester.ac.uk](mailto:js660@leicester.ac.uk), [aw24@leicester.ac.uk](mailto:aw24@leicester.ac.uk), [lfc11@leicester.ac.uk](mailto:lfc11@leicester.ac.uk), chp5@leicester.ac.uk)

b Centre for Environmental Health and Sustainability, University of Leicester, University Road, Leicester, LE1 7RH, United Kingdom ([sph22@leicester.ac.uk](mailto:sph22@leicester.ac.uk), ah618@leicester.ac.uk)

c Toxicology, UK Health Security Agency. Harwell Campus, Chilton, Didcot, OX11 0RQ, United Kingdom

d NIHR Health Protection Research Unit in Environmental Exposures and Health at the University of Leicester, University Road, Leicester, LE1 7RH, United Kingdom

e NIHR Leicester Biomedical Research Centre, Leicester General Hospital, Leicester LE5 4PW, United Kingdom

**Corresponding author:**

Professor Anna Hansell. Email: ah618@leicester.ac.uk

Centre for Environmental Health and Sustainability, University of Leicester, Leicester, United Kingdom

**Supplementary Data**

**MATERIALS AND METHODS**

**Site location and air sample collection**

Leicester (52^o^38’ N 1^o^5’ W) is located in the East Midlands of England, UK. It is an industrial city with a population of approximately 368,600 in 2021^1^ and is located about 90km from the coast. The city is predominantly surrounded by agricultural land.^2^

Sampling of airborne fungal spores was conducted continuously from 1^st^ March to 30^th^ November, from 2007-2020. Counting was not conducted in the winter months as airborne fungal spore levels are very low during this period. We excluded 2016 due to insufficient data. A 7-day volumetric spore trap (Burkard Manufacturing Co. Ltd.), situated 12m above ground level (60m above sea level) on the roof of the Bennett building, University of Leicester campus, was used throughout the study. This is a characteristically urban area, next to 69 acres of open parkland with tree lined avenues.

Sample slides were prepared according to standard procedures.^3-6^ Twenty-three fungal spore types were identified by light microscopy (Eclipse Ci, Nikon or AxioScope A1, Carl Zeiss), 18 to genus level. Spores were identified with reference to published articles^7-9^ and type slides donated by MAARA. Some fungal spores were counted jointly or as groups because visual distinction was not possible between genera, e.g., *Aspergillus* sp. and *Penicillium* sp., or using groupings based on similar morphology, as for ascospores, hyaline basidiospores, coloured basidiospores and “rusts and smuts”. Spores were counted along one central transverse under x600 or x630 magnification, and expressed as number of spores/m^3^ of air averaged over 24 hours.

Sampling of airborne grass pollen was carried out as described by Pashley *et al*^2^ using the same 7-day volumetric spore trap (Burkard Manufacturing Co. Ltd.) used for sampling fungal spores or an adjacent, identical trap located approximately 5m away.

**Antihistamine Prescribing Data**

Antihistamine prescribing data for Leicestershire was obtained from the OpenPrescribing website.^10^ The monthly data received was independently averaged for the 5 years available.

**Weather data**

Minimum and maximum temperature, precipitation, wind speed and direction were provided by Leicester city council air quality group. The meteorological station was located 5km from the trap site. The hourly data received was independently averaged to provide the daily weather data or summed in the case of precipitation to give total daily precipitation. Directional wind averages were calculated using circular means.

**Analysis**

Annual cumulative concentrations were calculated from the summation of daily average spore concentrations from 1^st^ March to 30^th^ November of each year for each spore type identified (Table S1). Here we present the eight most abundant fungal taxa, identified to genus level, plus coloured basidiospores (highlighted in Table S1). The groupings of ascospores and hyaline basidiospores, although highly abundant, were too heterogeneous for further analysis. All of the 9 spore types analysed have been implicated in fungal allergy.

The 90% method^4^ was used to define the spore season retrospectively, where the start date of the season is defined as the date when 5% of the total spore concentration is exceeded and the end when 95% of the total spore concentration is reached. The Seasonal Spore Integral (SSIn), which is the sum of the daily spore concentrations recorded during the defined spore season, was then calculated. Allergenic thresholds for *Alternaria* sp. and *Cladosporium* sp. were defined as 100 and 3000 spores/m^3^ respectively.^11-13^

Multiple regression analysis was used to examine the relationship between daily spore concentrations and selected meteorological parameters within fungal spore seasons for each taxon investigated. Lags of up to 3 days for meteorological variables were incorporated. Meteorological variable selection per model was performed using best subset selection with cross-validation, rather than metrics that penalise upon the addition of further variables. Spore counts were transformed using a logarithmic transformation (+1) as linear regression residuals were non-normal. Missing counts were imputed using interpolation from periods up to 7 days pre and post missing values, or the mean of surrounding years. Meteorological variables in the model included daily maximum air temperature (Tm, ^o^C), wind speed (WS, m/s), sum of precipitation (P, mm), and mean circular wind direction (WD, degrees). Wind direction was discretised into 4 categories (Q1: north east, Q2: south east, Q3: south west, Q4: north west). For each taxon run, the model providing the lowest root mean square error (RMSE) in prediction using k-fold cross-validation (k = 10) was selected as the final model per fungal spore group. Long-term trends were explored using linear regression across the 13-years of SSIn. An alpha value of 0.05 was used as the threshold for statistical significance.

All analyses were performed using GraphPad Prism version 7.05.237 for Windows (GraphPad Software, San Diego, Ca), Microsoft Excel 365, and R version 4. 1. 2.

**REFERENCES**

1. Office for National Statistics. Population and household estimates, England and Wales: Census 2021. 2022, June 28; <https://www.ons.gov.uk/peoplepopulationandcommunity/populationandmigration/populationestimates/datasets/populationandhouseholdestimatesenglandandwalescensus2021>.

2. Pashley CH, Fairs A, Edwards RE, Bailey JP, Corden JM, Wardlaw AJ. Reproducibility between counts of airborne allergenic pollen from two cities in the East Midlands, UK. *Aerobiologia.* 2009;25(4):249-263.

3. Newson R, Strachan D, Corden J, Millington W. Fungal and other spore counts as predictors of admissions for asthma in the Trent region. *Occupational and Environmental Medicine.* 2000;57(11):786-792.

4. Skjoth CA, Damialis A, Belmonte J, et al. Alternaria spores in the air across Europe: abundance, seasonality and relationships with climate, meteorology and local environment. *Aerobiologia.* 2016;32(1):3-22.

5. Bednarz A, Pawłowska S. A fungal spore calendar for the atmosphere of Szczecin, Poland. *Acta agrobotanica.* 2016;69(3).

6. Sadys M, Adams-Groom B, Herbert RJ, Kennedy R. Comparisons of fungal spore distributions using air sampling at Worcester, England (2006-2010). *Aerobiologia.* 2016;32(4):619-634.

7. Smith EG. *Sampling and identifying allergenic pollens and molds. Volume II. An illustrated identification manual for air samplers.* Vol II: Blewstone Press; 1986.

8. Lacey M, West J. *The air spora : a manual for catching and identifying airborne biological particles.* Dordrecht: Springer; 2006.

9. Levetin E, Horner WE, Scott JA. Taxonomy of Allergenic Fungi. *J Allergy Clin Immunol Pract.* 2016;4(3):375-385.e371.

10. Bennett Institute for Applied Data Science UoO. OpenPrescribing. 2024; <https://openprescribing.net/>.

11. Bagni N, Davies KR, Mallea M, Nolard N, Spieksma FT, Stix E. Sporenkonzentrationen in Städten der Europäischen Gemeinschaft (EG): II. Cladosporium‐ und Alternaria‐Sporen. *Acta allergologica.* 1977;32(2):118-138.

12. Gravesen S. Fungi as a Cause of Allergic Disease. *Allergy (Copenhagen).* 1979;34(3):135-154.

13. Rapiejko P, Stanlaewicz W, Szczygielski K, Jurkiewicz D. [Threshold pollen count necessary to evoke allergic symptoms]. *Otolaryngol Pol.* 2007;61(4):591-594.

**Table S1.** Characteristics of fungal spore distribution for all spore types identified.

| Spore Type | Mean yearly spore concentration [March - Nov] (spores/m^3^) | Mean yearly spore concentration [March - Nov] SD | % Total spore concentration | Mean Daily Maximum Peak (spores/m^3^) | Greatest Maximum Daily Peak (spores/m^3^) | Smallest Maximum Daily Peak (spores/m^3^) |
| --- | --- | --- | --- | --- | --- | --- |
| ***Cladosporium*** **†** | 875,395 | 236,641 | 26.23 | 38,164 | 70,029 | 18,042 |
| Ascospores | 620,921 | 228,623 | 18.61 | 19,986 | 40,047 | 8,333 |
| ***Sporobolomyces*** **†** | 485,845 | 260,066 | 14.56 | 50,388 | 139,446 | 8,382 |
| Hyaline basidiospores | 422,520 | 189,502 | 12.66 | 12,200 | 33,757 | 4,186 |
| ***Tilletiopsis* †** | 312,306 | 315,565 | 9.36 | 30,124 | 89,274 | 1,362 |
| **Coloured basidiospores †** | 136,452 | 32,187 | 4.09 | 3,967 | 8,455 | 2,654 |
| ***Didymella* †** | 104,532 | 109,978 | 3.13 | 8,035 | 27,342 | 967 |
| ***Leptosphaeria* †** | 36,495 | 18,193 | 1.09 | 1,426 | 2,440 | 465 |
| ***Aspergillus/***  ***Penicillium* type †** | 34,715 | 9,754 | 1.04 | 1,272 | 2,715 | 496 |
| ***Ustilago* †** | 32,008 | 10,325 | 0.96 | 4,165 | 8,375 | 1,776 |
| ***Alternaria* †** | 25,353 | 10,258 | 0.76 | 1,655 | 3,700 | 918 |
| *Ganoderma* | 13,920 | 3,995 | 0.42 | 307 | 420 | 161 |
| Rusts/smuts | 13,870 | 6,197 | 0.42 | 448 | 724 | 220 |
| *Botrytis* | 12,797 | 5,242 | 0.38 | 460 | 1,190 | 248 |
| *Entomophthora* | 5,287 | 2,114 | 0.16 | 200 | 372 | 99 |
| *Epicoccum* | 3,876 | 1,145 | 0.12 | 186 | 397 | 79 |
| *Erysiphe* | 3,604 | 1,487 | 0.11 | 117 | 180 | 67 |
| *Polythrincium* | 2,499 | 1,241 | 0.07 | 135 | 337 | 30 |
| *Lewia/Pleospora* | 2,492 | 976 | 0.07 | 173 | 459 | 43 |
| *Torula* | 1,590 | 414 | 0.05 | 81 | 161 | 43 |
| *Stemphylium* | 1,282 | 1,459 | 0.04 | 191 | 620 | 24 |
| *Drechslera* | 353 | 193 | 0.01 | 43 | 140 | 12 |
| *Pithomyces* | 247 | 165 | 0.01 | 30 | 50 | 12 |

Fungal spores were categorised to genus level when applicable, otherwise spores were grouped by visual distinction. Mean concentrations were calculated yearly from March to November and averaged across the 13 years of data collection. Maximum daily peak was calculated yearly as the highest number of spores recorded in a day for each spore type, during the recording period (March to November). Spore types in bold and indicated with **†** were included in further analysis, as they exceeded a mean daily maximum peak of 1000 spores/m^3^.

**Table S2** Seasonal characteristics of the main spore types studied

SSIn – seasonal spore integral (i.e., total spore concentration during the spore season)

Spore season duration - defined by the 90% method, where the start of the season is the date at which 5% of the total spore concentration for the 9 months was recorded and 95% being the end date of the season and is based on 13 years of data.

**
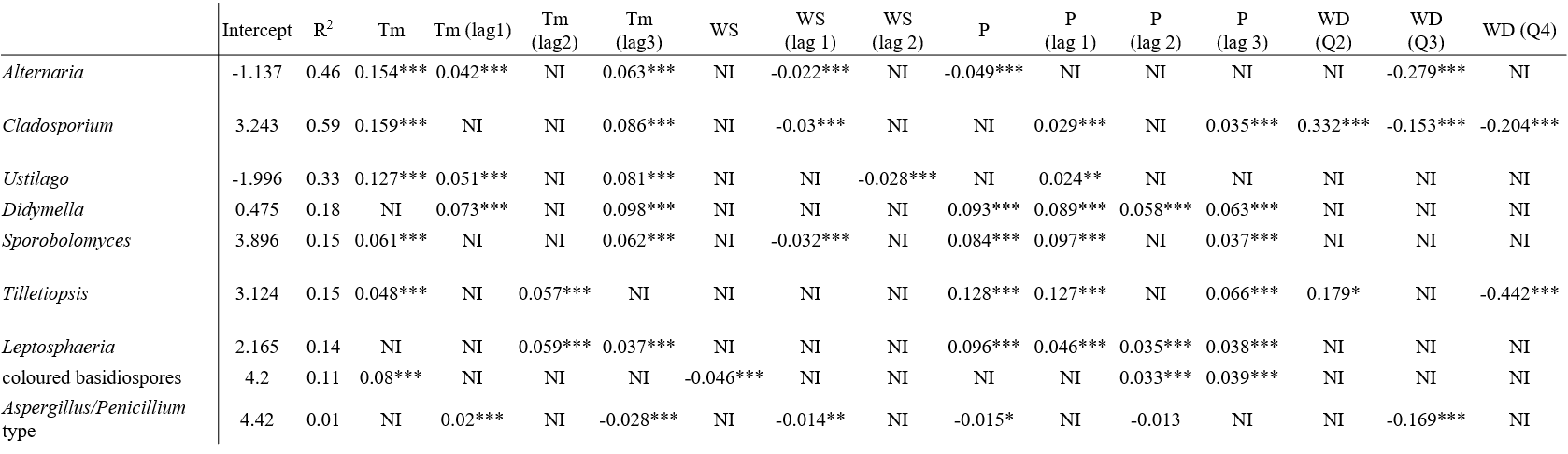
**

*p<0.1; **p<0.05; ***p<0.01

NI = variable not included in final model, R^2^ = R-squared value, Tm = Maximum daily temperature
(^o^C), WS = maximum daily wind speed (m/s), P = daily precipitation (mm), WD = wind direction (quadrant, Q) (Q1: north east, Q2: south east, Q3: south west, Q4: north west)

**Table S3 Multiple regression analysis of the effects of meteorological variables on**

**various fungal spore concentrations.**

**Table S4** Linear regression trend analysis for annual changes in Seasonal Spore Integral

(SSIn)

|  | **Slope** | **R^2^** | **Deviation from zero** |
| --- | --- | --- | --- |
| *Cladosporium* | 5751 ± 14866 | 0.01342 | ns |
| *Alternaria* | 1352 ± 505.2 | 0.3944 | P = 0.02 |
| *Didymella* | -11365 ± 6014 | 0.2451 | P = 0.09 |
| *Sporobolomyces* | -32819 ± 13137 | 0.3620 | P = 0.03 |
| *Tilletiopsis* | -37315 ± 16506 | 0.3172 | P = 0.05 |
| *Ustilago* | -362.1 ± 656.9 | 0.02688 | ns |
| *Leptosphaeria* | -1741 ± 1020 | 0.2093 | ns |
| *Asp/Pen* type | 465.9 ± 600.1 | 0.05195 | ns |
| Coloured basidiospores | 616 ± 2036 | 0.008254 | ns |
| Total spores | -105641 ± 42813 | 0.3563 | P = 0.03 |

SSIn – seasonal spore integral (i.e., total spore concentration during the spore season as defined by the 90% method)
